# HDAC2 Regulates Glial Cell Activation in Ischemic Mouse Retina

**DOI:** 10.3390/ijms20205159

**Published:** 2019-10-17

**Authors:** Mi Sun Sung, Hwan Heo, Gwang Hyeon Eom, So Young Kim, Helong Piao, Yue Guo, Sang Woo Park

**Affiliations:** 1 Department of Ophthalmology, Chonnam National University Medical School and Hospital, Gwangju 61469, Korea; sms84831@hanmail.net (M.S.S.); intereye@empal.com (H.H.); syou8246@naver.com (S.Y.K.); 179302143long@gmail.com (H.P.); gyhappypot@gmail.com (Y.G.); 2 Department of Pharmacology, Chonnam National University Medical School, Hwasungun 58128, Korea; eomgh@chonnam.ac.kr

**Keywords:** histone deacetylase, glial cell, ischemia, retina, retinal ganglion cell

## Abstract

The current study was undertaken to investigate whether histone deacetylases (HDACs) can modulate the viability of retinal ganglion cells (RGCs) and the activity of glial cells in a mouse model of retinal ischemia-reperfusion (IR) injury. C57BL/6J mice were subjected to constant elevation of intraocular pressure for 60 min to induce retinal IR injury. Expression of macroglial and microglial cell markers (GFAP and Iba1), hypoxia inducing factor (HIF)-1α, and histone acetylation was analyzed after IR injury. To investigate the role of HDACs in the activation of glial cells, overexpression of HDAC1 and HDAC2 isoforms was performed. To determine the effect of HDAC inhibition on RGC survival, trichostatin-A (TSA, 2.5 mg/kg) was injected intraperitoneally. After IR injury, retinal GFAP, Iba1, and HIF-1α were upregulated. Conversely, retinal histone acetylation was downregulated. Notably, adenoviral-induced overexpression of HDAC2 enhanced glial activation following IR injury, whereas overexpression of HDAC1 did not significantly affect glial activation. TSA treatment significantly increased RGC survival after IR injury. Our results suggest that increased activity of HDAC2 is closely related to glial activation in a mouse model of retinal IR injury and inhibition of HDACs by TSA showed neuroprotective potential in retinas with IR injuries.

## 1. Introduction

The acetylation of histone N-terminal tails controls the interaction between histones and DNA in chromatin and results in chromatin remodeling, which is a key step in transcriptional regulation [1,2]. For example, hyperacetylation is associated with transcriptional activation, whereas deacetylation is associated with transcriptional repression. The equilibrium between the activity of histone acetyltransferases (HAT) and histone deacetylases (HDAC) is tightly regulated in neurons under normal conditions. The impairment of acetylation homeostasis results in a shift toward deacetylation, which is a frequent molecular feature of neurodegenerative diseases [3,4]. The inhibition of HDACs has been attempted to restore homeostatic conditions in such diseases. For example, the application of valprioc acid, a class I and IIa HDAC inhibitor, or trichostatin A (TSA), a pan-HDAC inhibitor, was reported to be neuroprotective in retinal ganglion cells (RGC) in an animal model of optic nerve crushing, retinal ischemia, and chronic ocular hypertension [5,6,7,8].

RGC can be damaged by various ocular diseases, such as retinal vein or artery occlusion, diabetic retinopathy and optic neuropathies including glaucoma [9,10,11]. RGC can be damaged via multiple stimuli, such as ischemia, oxidative stress, excitotoxicity, excessive production of nitric oxide, elevation of intraocular pressure (IOP), or neurotrophic factor deprivation [12,13,14,15]. It is well known that glial cells, like Müller cells, astrocytes, or microglia, are activated under retinal ischemic conditions and activated glial cells may facilitate the neurodestructive changes in ischemic retina [16,17]. In terms of regulating glial cell function, Chen et al. [18]. reported that inhibition of HDAC can attenuate microglial activation in the brain. Kanski et al. [19]. reported that histone acetylation can modulate glial fibrillary acidic protein (GFAP) expression and reorganize the intermediate filaments in astrocytes. Among the various HDAC isoforms, previous studies have demonstrated that HDAC1, 2, 3, and 6 are the major contributors to total HDAC activity in rodent retina and, importantly, the increased retinal HDAC activity induced by retinal ischemia may mostly attributed to increased HDAC1 and 2 activity [20,21].

The current study was undertaken to investigate the role of HDACs on RGC survival and glial cell activation in retinas with ischemia-reperfusion (IR) injuries and the mechanisms by which the inhibition of HDAC activity can increase RGC survival in retinas with IR injuries.

## 2. Results

### 2.1. Expression of GFAP, Iba1, HIF-1α, and Acetyl-H3 in Retinas after IR Injury

**Figure 1:**
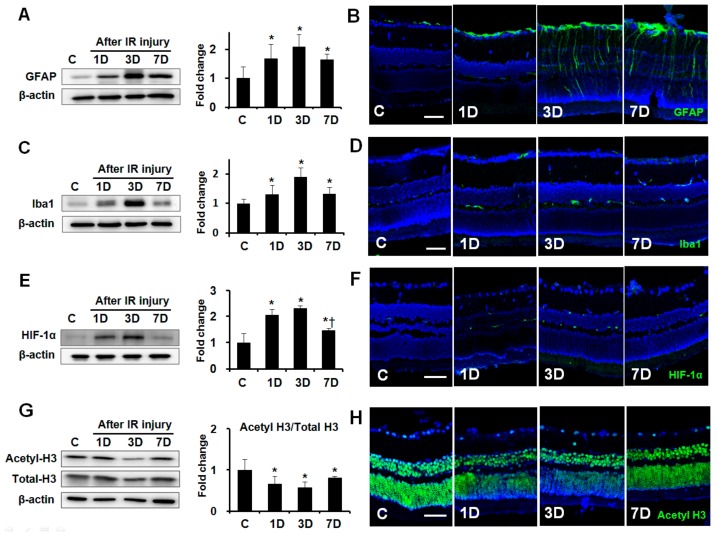
Expression of GFAP, Iba1, HIF-1α, and acetyl-H3 in ischemic retinas according to time interval. Representative cropped western blots depicting GFAP, Iba1, HIF-1α, acetyl-H3, and total-H3 protein levels, respectively. Full size images are presented in Supplementary Figure S1. GFAP (**A**), Iba1 (**C**), and HIF-1α (**E**) expression peaked at 3 days after IR injury. In contrast, the level of acetyl-H3 expression was downregulated at 1, 3, and 7 days after IR injury (**G**). Immunohistochemistry for GFAP (**B**), Iba1 (**D**), and HIF-1α (**F**) show markedly upregulated expression at 3 days after IR injury. Immunohistochemical staining of acetyl-H3 shows that staining intensity markedly decreased in the ganglion cell layer and outer nuclear layer at 3 days after IR injury (**H**). Relative chemiluminescence intensity for each protein band was normalized using β-actin as a calibrator. Error bars, SD (*n* = 6 retinas/group). Scale bars, 50 µm. * *p* < 0.01 compared to the control group. † *p* < 0.01 compared to the 3D group. All comparisons were performed using one-way analysis of variance (ANOVA) with post hoc Bonferroni’s test.

To evaluate the expression of GFAP, ionized calcium binding adaptor molecule-1(Iba1), hypoxia inducing factor (HIF)-1α, and the extent of histone acetylation after retinal IR injury, retinas were removed from the eyes at 1, 3, and 7 days after IR injury (*n* = 6 retinas per time point, Figure 1). Western blot and immunohistochemical analysis showed that GFAP and Iba1 expression peaked at 3 days after IR injury relative to the control (2.08 ± 0.42-fold and 1.90 ± 0.31-fold, respectively; both *p* < 0.01, Figure 1A–D). HIF-1α expression peaked at 3 days after retinal ischemia relative to the control (2.32 ± 0.10-fold; *p* < 0.01, Figure 1E,F), and its upregulation remained significant for all the following time points after IR injury. In contrast, the level of acetylated-histone H3 (acetyl-H3) expression was downregulated at 1, 3, and 7 days after IR injury relative to the control (0.66 ± 0.19-fold, 0.57 ± 0.08-fold, and 0.82 ± 0.03-fold, respectively; all *p* < 0.01, Figure 1G,H), with a minimum at 3 days in ischemic retinas.

### 2.2. Effect of TSA on Expression of GFAP, Iba1, HIF-1α, and Acetyl-H3 in Retinas after IR Injury

**Figure 2:**
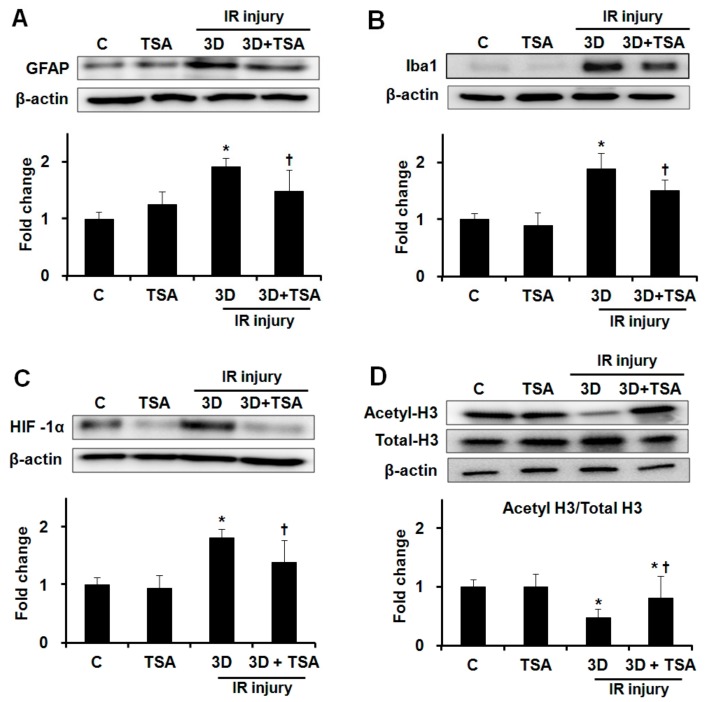
Effect of TSA on expression of GFAP, Iba1, HIF-1α, and acetyl-H3 in ischemic retinas. Representative cropped western blots depicting GFAP, Iba1, HIF-1α, acetyl-H3, and total-H3 protein levels, respectively. Full size images are presented in Supplementary Figure S2. TSA treatment significantly inhibited increased expression of GFAP (**A**), Iba1 (**B**), and HIF-1α (**C**) in ischemic retinas after IR injury. TSA treatment prevented the downregulation of acetyl-H3 expression in ischemic retinas relative to saline treatment after IR injury (**D**). Relative chemiluminescence intensity for each protein band was normalized using β-actin as a calibrator. Error bars, SD (*n* = 10 retinas/group). * *p* < 0.01 compared to the control group. † *p* < 0.01 compared to the saline-treated 3D group. All comparisons were performed using one-way ANOVA with post hoc Bonferroni’s test.

We evaluated whether TSA treatment would affect the expression of GFAP, Iba1, HIF-1α, and acetyle-H3 after IR injury. TSA treatment significantly inhibited GFAP, Iba1, and HIF-1α expression in ischemic retinas at 3 days after IR injury (1.92 ± 0.22 vs. 1.48 ± 0.48-fold, 1.89 ± 0.38 vs. 1.51 ± 0.29-fold, and 1.82 ± 0.17 vs. 1.39 ± 0.49-fold, respectively; all *p* < 0.01, *n* = 10 retinas/group, Figure 2–C). TSA treatment prevented the downregulation of acetyl-H3 expression in ischemic retinas at 3 days after IR injury (0.48 ± 0.14 vs. 0.82 ± 0.37-fold; *p* < 0.01, *n* = 10 retinas/group, Figure 2D).

### 2.3. Effect of Overexpression of HDAC1 and HDAC2 on Glial Cell Activity and iNOS Expression

**Figure 3:**
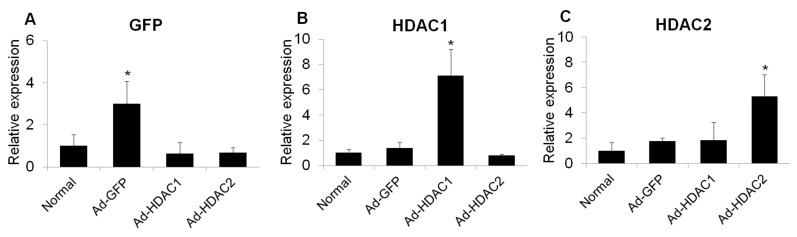
The mRNA expression of GFP (**A**), HDAC1 (**B**), and HDAC2 (**C**) in retinas at 3 days after intravitreal injection of adenovirus. Data were normalized to the expression of 18S-rRNA. Error bars, SD (*n* = 10 retinas/group). * *p* < 0.001 compared to the normal retina. All comparisons were performed using one-way ANOVA with post hoc Bonferroni’s test.

To determine whether HDAC activity affects glial activation in mouse retinas, we overexpressed HDAC1 and HDAC2 by adenoviral transduction and evaluated the mRNA levels of GFAP, Iba1 and inducible nitric oxide synthase (iNOS) at 6 days after intravitreal injection. Three days after intravitreal injection of adenoviral green fluorescent protein (GFP), HDAC1, and HDAC2, the expression of GFP, HDAC1, and HDAC2 increased significantly (Figure 3).

**Figure 4:**
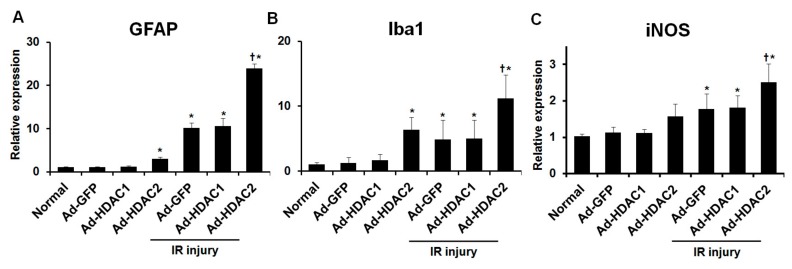
The effect of overexpression of HDAC1 and HDAC2 on glial cells and iNOS activity in retinas. By adenoviral transduction, overexpression of HDAC2 significantly increased the expression of GFAP and Iba1 mRNA in eyes with and without IR injury compared to eyes with overexpression of GFP (**A**,**B**). Overexpression of HDAC2 significantly increased iNOS mRNA expression in eyes with IR injury compared to eyes with overexpression of GFP (**C**). Overexpression of HDAC1 did not significantly change the expression of GFAP, Iba1, and iNOS mRNA in eyes with and without IR injury compared to eyes with overexpression of GFP. Data were normalized to the expression of 18S-rRNA. Error bars, SD (*n* = 10 retinas/group). * *p* < 0.001 compared to the normal group. † *p* < 0.001 compared to the Ad-GFP + IR injury group. Comparisons were performed using unpaired 2-tailed Student’s *t*-test.

**Figure 5:**
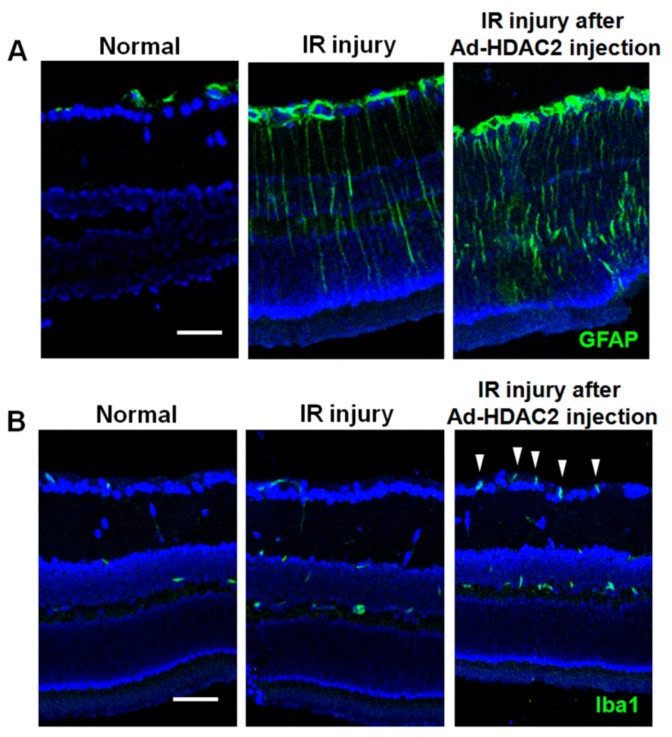
The effect of HDAC2 overexpression on glial cell activities at 3 days after IR injury. IR injury markedly upregulated the expression of macroglial (GFAP; **A**) and microglial (Iba1; **B**) expression at day 3. The increased expression of Iba1 was remarkable at the ganglion cell layer and nerve fiber layer (white arrowheads). Overexpression of HDAC2 significantly exacerbates glial cell expressions in mouse retinas after IR injury. Scale bars, 50 µm.

In eyes without IR injury, eyes with overexpression of HDAC2 showed significant upregulation of the expression of GFAP and Iba1 mRNA relative to eyes with overexpression of GFP (1.07 ± 0.10 vs. 2.96 ± 0.50-fold and 1.24 ± 0.87 vs. 6.32 ± 1.95-fold, respectively; both *p* < 0.001, *n* = 10 retinas/group, Figure 4A,B). We found that IR injury significantly increased the expression of GFAP and Iba1 mRNA in mouse retinas. In eyes with IR injuries, significant upregulation of the expression of GFAP and Iba1 mRNA was found in eyes with overexpression of HDAC2 relative to eyes with overexpression of GFP (10.10 ± 1.22 vs. 23.85 ± 1.10-fold and 4.87 ± 2.95 vs. 11.19 ± 3.64-fold, respectively; both *p* < 0.001, *n* = 10 retinas/group, Figure 4A,B). Similarly, in eyes with IR injuries, significant upregulation of the expression of iNOS mRNA was found in eyes with overexpression of HDAC2 compared to eyes with overexpression of GFP (1.78 ± 0.41 vs. 2.51 ± 0.51-fold; *p* < 0.001, *n* = 10 retinas/group, Figure 4C). However, in eyes with and without IR injuries, overexpression of HDAC1 did not produce significant changes in GFAP, Iba1 and iNOS mRNA expression compared to eyes with overexpression of GFP (*n* = 10 retinas/group, Figure 4A–C). Figure 5 shows the results of immunohistochemical stain for GFAP and Iba1 after IR injury in eyes with and without HDAC2 overexpression. Overexpression of HDAC2 exacerbates microglial and macroglial activation in mouse retinas after IR injury.

### 2.4. Effect of TSA on RGC Loss in Retina after IR Injury

**Figure 6:**
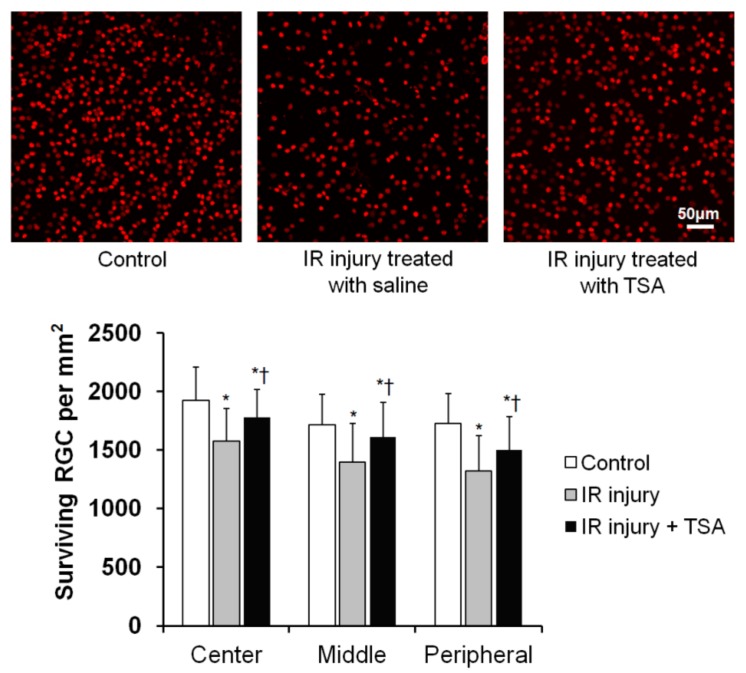
The effect of TSA on RGC survival in ischemic retinas. Retinal flat mounts are shown for the control, saline-treated ischemic retina, and TSA-treated ischemic retina groups (*n* = 10 retinas/group). Quantitative analysis of RGC survival are also shown as graph. Scale bars, 50 µm. * *p* < 0.05 compared to the control group. † *p* < 0.05 compared to the saline-treated IR injury group. All comparisons were performed using one-way ANOVA with post hoc Bonferroni’s test.

RGC survival was evaluated by immunohistochemistry of retinal whole mounts with anti-Brn3a antibody labeling. There was a significant difference in RGC density between TSA-treated and saline-treated retinas at 2 weeks after IR injury. Saline-treated retinas had significant RGC losses of approximately 17.9% in the center, 18.7% in the middle, and 23.4% in the peripheral area compared to the control retinas. TSA treatment prevented RGC loss in the center, middle, and peripheral area relative to saline-treated retinas (all *p* < 0.05, *n* = 10 retinas/group, Figure 6, Table 1). These results suggest that TSA has a neuroprotective effect on RGCs after IR injury.

## 3. Discussion

In terms of experimental retinal ischemia, it is well known that short-term IOP elevation results in neuronal cell death especially in the inner retina [9,22]. On the contrary, glial cells in the retina, like Müller cells or astrocytes, are activated under similar retinal ischemic conditions [16]. Furthermore, activated astroglial cells may participate in neuronal damage and are characterized by upregulation of GFAP and increased production of inflammatory mediators [17,23,24]. The acute IOP elevation that was used in our study is well suited as a reproducible model for investigation of mechanisms of neuronal degeneration in the inner retina. Among diverse post-translational modifications, intracellular acetylation homeostasis, which is controlled by HAT and HDAC, plays a significant role in the regulation of cellular activity by epigenetic modulation of gene expression and acetylation status of non-histone proteins [25]. The regulation of HDAC has been associated with important cellular functions such as gene expression, cell differentiation, maintenance of cellular homeostasis, apoptosis and wound healing [3,26].

Our results showed that pharmacologic inhibition of HDACs can protect RGCs and, interestingly, that the overexpression of HDAC2 is closely related to increased glial cell activity in ischemic retina. We observed a significant increase in the levels of GFAP, Iba1, and iNOS mRNA in ischemic retinas with HDAC2 overexpression. On the other hand, HDAC1 overexpression had no effect on GFAP and iNOS mRNA expression in ischemic retinas.

Previous studies have shown that increased retinal HDAC activity contributes to retinal degeneration in retinal ischemia, chronic ocular hypertension and optic nerve damage [5,6,7,8,27,28]. Among class I HDAC isoforms, which have been known to be expressed in the retina, Fan et al. [29]. reported that HDAC1 and HDAC2 expression increased in ischemic retinas, and HDAC3 expression did not significantly change after retinal ischemia. Accordingly, the selective inhibition of HDAC2 was reported to significantly protect the retina from ischemic injury as effectively as non-selective HDAC inhibitors [20].

Previous studies have shown that increased activity of HDAC2 is associated with cancers, diverse neurodegenerative diseases, and neural toxicity [30,31,32,33]. In addition, HDAC2 can negatively regulate neuronal functions such as synaptic plasticity, spine density, learning, and memory formation [30]. These findings have led us to believe and also demonstrate that HDAC2 activity plays a significant role in neurodegenerative diseases, and that HDAC2 may be a therapeutic target for neurodegenerative disorders. Recently, an inhibitory effect of HDACs on glial activation has been suggested in diverse neurodegenerative disorders. The application of non-selective HDAC inhibitors suppressed the expression of tumor necrosis factor-alpha (TNF-α), interleukin-6 (IL-6) and heat-shock protein 70 (HSP70) in microglia or astrocyte activated with lipopolysaccharide (LPS) [34,35,36]. Similar to previous studies, Faraco et al. [37]. reported that the inhibition of HDACs is essential for suppression of pro-inflammatory mediators such as iNOS, COX2 and IL-1β and can boost the anti-inflammatory effects of steroid treatment on LPS-activated glial cells in vitro and in vivo. Durham et al. [38] reported that pharmacologic inhibition or selective knockdown of HDAC1 or HDAC2 can suppress the expression of the cytokines IL-6 and TNF-α in LPS-activated murine microglial cells. Notably, they reported that knockdown of HDAC1 did not produce any change in the response to LPS compared to control microglial cells, while knockdown of HDAC2 resulted in reduced induction of IL-6 and TNF-a expression in response to LPS in microglial cells.

It is of particular interest that HDAC1 and HDAC2 may have different regulating effects on cellular activation despite a very high sequence similarity and functional redundancy between these two isoforms [39,40]. Previously, we reported that a significant increase in the level of α-smooth muscle actin (α-SMA) mRNA and collagen deposition can be observed in conjunctiva with HDAC1 overexpression, while HDAC2 overexpression had no effect on α-SMA mRNA expression and collagen density in a rat trabeculectomy model [26]. Taken together, we suggest that acetylation-dependent transcriptional regulation for retinal glial cell activation can be HDAC isoform-specific.

Nevertheless, the exact mechanisms of the HDACs on the regulation of glial cell activity have yet to be clarified. In this study, we do not provide target proteins regulated by HDAC2 for promoting glial cell activation in ischemic retinas. Regarding this issue, previous studies have suggested that inhibition of the pro-inflammatory response of glial cells can be attributed to suppression of transcriptional activation by HDAC inhibition within glial cells without translation of anti-inflammatory proteins [37,38]. Further studies will be needed to identify potential target proteins that are implicated in the activation of retinal glial cells by HDAC2.

One of the limitations of our study is that we did not evaluate the RGC function. RGCs undergo functional impairment prior to morphological changes after IR injury [41]. In addition, structural restoration of the RGCs does not necessarily mean its functional restoration. Therefore, it is unclear whether TSA is also effective for the functional restoration of RGC after IR injury. However, our results demonstrated that TSA significantly reduce retinal histological damage and RGC loss following IR injury, and we believe that our findings are important for future neuroprotective therapeutic approach in various retinal diseases. Future investigations regarding the functional efficacy of TSA would be needed before its clinical application.

## 4. Materials and Methods

### 4.1. Animal Use

All experiments were conducted in accordance with the Association for Research in Vision and Ophthalmology (ARVO) Statement for the Use of Animals in Ophthalmic and Vision Research. The protocol was approved by the Institutional Animal Care and Use Committee of Chonnam National University Hospital (Permission code, CNU IACUC-H-2015-31, 9 May 2015). C57BL/6J mice, obtained at 3 months of age (20–25 g in weight), were housed in individual cages under controlled lighting conditions (12 h light/12 h dark) and given tap water and food ad libitum throughout the duration of the study.

### 4.2. Retinal Ischemia-Reperfusion (IR) Injury

**Figure 7:**
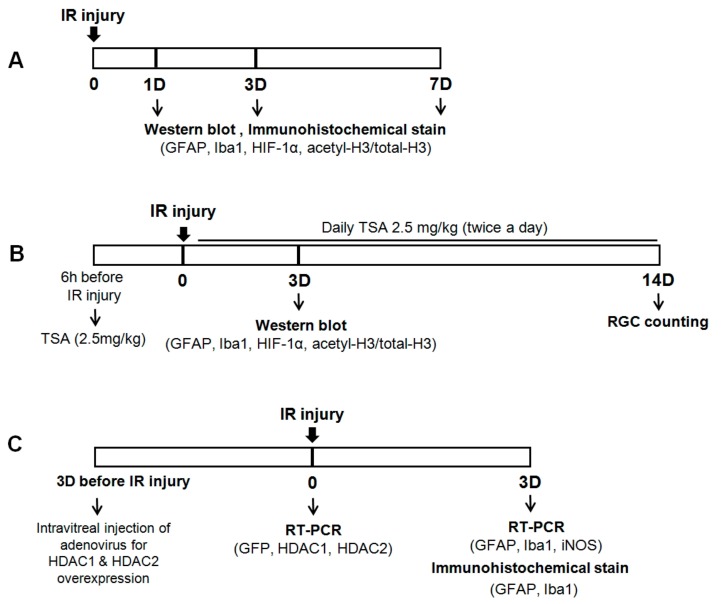
Schematic representation of experimental protocols in this study. Each arrow indicates a procedural time point. (**A**) To investigate retinal changes after IR injury, retinal tissue was obtained at 1, 3, and 7 days after IR injury. (**B**) To investigate the effect of TSA after IR injury, TSA (2.5 mg/kg) was injected intraperitoneally 6 h before retinal IR injury and twice daily after retinal IR injury. Retinal tissue was obtained at 3 and 14 days after IR injury. (**C**) To evaluate whether HDACs activity is associated with glial cell activity, HDAC1 and HDAC2 adenovirus was intravitreally injected 3 days before IR injury. Retinal tissue was obtained at 3 days after IR injury.

For inducing retinal ischemia, C57BL/6J mice were anesthetized with a mixture of ketamine hydrochloride (40 mg/kg; Yuhan, Seoul, Korea) and xylazine hydrochloride (4 mg/kg, Rompun, Bayer Korea, Seoul, Korea) by intramuscular injection. A cannula was inserted into the anterior chamber connected by flexible tubing to a reservoir. By raising the reservoir, we continuously increased IOP above systolic blood pressure (90–100 mmHg) over 60 min. Ischemia was confirmed by blanching of the iris vessels or the loss of the retinal red reflex. After removing the cannula, reperfusion of the retinal vasculature was checked by examining the fundus. The sham operations were performed in the eyes of the control group, but without IOP elevation. We performed 3 different types of experiments with the mice in this study (Figure 7).

### 4.3. Adenoviral GFP, HDAC1, and HDAC2

To evaluate whether HDACs activity is associated with glial cell activity in mouse retina, HDAC1 and HDAC2 adenoviruses, purchased from Applied Biological Materials (096643A and 096646A; Richmond, BC, Canada), were used for the overexpression of HDAC1 and HDAC2. Adenoviral GFP was used as a control vector. Infection of adenoviral GFP, HDAC1, and HDAC2 was confirmed by real-time polymerase chain reaction (RT-PCR). The eyes were dilated with one drop of a combination of 0.5% tropicamide and 0.5% phenylephrine (Mydrin-P; Santen, Osaka, Japan), and 1.5μL of viral solution (1 × 10^10^ pfu) was injected into the vitreous cavity using a Hamilton syringe (Hamilton Company, Reno, NV, USA) at 3 days before IR injury. Adenoviral titer was determined using the Adeno-X Rapid Titer kit (Clontech Laboratories, Mountain View, CA, USA).

In this experiment, the eyes were divided into 7 groups: (1) eyes without viral injection; (2) eyes that underwent adenoviral GFP injection; (3) eyes that underwent adenoviral HDAC1 injection; (4) eyes that underwent adenoviral HDAC2 injection; (5) eyes with retinal IR injury that underwent adenoviral GFP injection; (6) eyes with retinal IR injury that underwent adenoviral HDAC1 injection; and (7) eyes with retinal IR injury that underwent adenoviral HDAC2 injection. In a mouse model of retinal IR injury, retinal ischemia was induced 3 days after intravitreal adenoviral injection. Topical antibiotic eye drops were administered four times a day in all eyes until the end of the experiments. All mice were euthanized 6 days after intravitreal injection.

### 4.4. Real-Time Polymerase Chain Reaction

The mRNA expression of gene encoding GFAP, Iba1 and iNOS was evaluated by RT-PCR. Total RNA from tissues was extracted using TRIzol Reagent (Invitrogen, Grand Island, NY, USA). cDNA was synthesized using an iScript cDNA Synthesis Kit (Bio-Rad Laboratories, Hercules, CA, USA) and analyzed by RT-PCR with QuantiTech SYBR Green RT-PCR Master Mix (Qiagen, Valencia, CA, USA) using the Rotor-Gene Q (Qiagen, Hilden, Germany). The primer sequences used in PCR were as follows: GFP, 5′-TGGTCCCAATTCTCGTGGAA-3′ (forward), 5′-CCTCTCC GCTGACAGAAAATT-3′ (reverse); HDAC1, 5′-TGGGGCTGGCAAAGGCAAGT-3′ (forward), 5′-G ACCACTGCACTAGGCTGGA-3′ (reverse); HDAC2, 5′-TGGGCTGGAGGACTACATCA-3′ (forward), 5′-CGGTCATCACGCGATCTGTT-3′ (reverse); GFAP, 5′-TCCTGGAACAGCAAAACAA G-3′ (forward), 5′-CAGCCTCAGGTTGGTTTCAT-3′ (reverse); Iba1, 5′-GGATCAACAAGCAATTC CTCGA-3′ (forward), 5′-CTGAGAAAGTCAGAGTAGCTGA-3′ (reverse); iNOS, 5′-CGAAACGCTT CACTTC CAA-3′ (forward), 5′-TGAGCCTATATTGCTGTGGCT-3′ (reverse). All data were normalized to the expression of 18S-rRNA.

### 4.5. Pharmacological Treatment

To investigate the neuroprotective effect of HDAC inhibitors in a mouse mode of retinal IR injury, mice were treated with a potent HDAC inhibitor, TSA. TSA (2.5 mg/kg) was injected intraperitoneally 6 h before retinal IR injury and twice daily after retinal IR injury. In this experimental protocol, mice were randomly divided into 4 groups as follows: (1) control group; (2) TSA group that received intraperitoneal TSA injection without retinal IR injury; (3) IR injury group that received intraperitoneal saline injection; and (4) IR injury + TSA group that received intraperitoneal TSA injection before and after retinal IR injury. The dosage and injection protocol of TSA in this study was based on a previous similar study [20]. Mice were euthanized on day 3 for western blot and immunohistochemical analysis and on day 14 for retinal wholemounts. The experiments were performed on three independent sets of mice.

### 4.6. Western Blot Analysis

For western blot analyses, whole retinas were used immediately or frozen at −70 °C until use. Retinal tissues were homogenized in a glass-teflon Potter homogenizer in lysis buffer (PRO-PREPTM, iNtRoN Biotechnology, Seoul, Korea). Each sample (10 µg) was separated in a 10% polyacrylamide mini-gel. After protein transfer, membranes were blocked for 1 h at room temperature in Tris-buffered saline–Tween-20 solution [TBS-T; 10 mM Tris–HCl (pH 7.6), 150 mM NaCl, and 0.1% Tween-20] containing 5% non-fat dry milk. After blocking, membranes were incubated overnight at 4 °C with a primary antibody against GFAP (1:3000; Cell Signaling Technology, Danvers, MA, USA), Iba1 (1:1000; Santa Cruz Biotechnology, Santa Cruz, CA, USA), HIF-1α (1:1000; Santa Cruz Biotechnology), total-histone H3 (total-H3) (1:1000; Cell Signaling Technology), acetyl-H3 (1:1000; Cell Signaling Technology), or β-actin (1:10,000; Sigma Missouri, USA). After three washes with TBS-T, the membranes were incubated for 1 h at room temperature with a peroxidase-conjugated goat anti-mouse IgG (1:3000; Santa Cruz Biotechnology) or peroxidase-conjugated goat anti-rabbit IgG (1:3000; Cell Signaling Technology) in TBS-T containing 5% non-fat dry milk. Signals were visualized by enhanced chemiluminescence and quantified using a LAS-3000 image analyzer (Fujifilm, Tokyo, Japan).

### 4.7. Immunohistochemical Analysis

Immunohistochemical staining of 7-µm wax sections of full-thickness retinas was performed by immunofluorescence with the following primary antibodies: mouse monoclonal anti-GFAP antibody (1:200; Cell Signaling Technology), mouse monoclonal anti-Iba1 antibody (1:150; Santa Cruz Biotechnology), mouse monoclonal anti-HIF-1α (1:200; Cell Signaling Technology), and rabbit monoclonal anti-acetyl-H3 (1:200; Cell Signaling Technology). Tissues were blocked with 1% bovine serum albumin in PBS for 1 h at room temperature to prevent nonspecific background staining, and then with primary antibodies overnight at 4 °C. The sections were washed several times, incubated with Alexa Fluor 488–conjugated chicken anti-mouse IgG (1:250; Invitrogen, Carlsbad California) or Alexa Fluor 546-conjugated rabbit anti-goat IgG (1:250; Invitrogen) for 4 h at 4 °C, and then washed again with PBS. The sections were counterstained with Hoechst 33342/PBS (0.1 µg/mL; Invitrogen). Images were analyzed using a Zeiss LSM 510 confocal microscope (Carl Zeiss, Jena, Germany).

### 4.8. Retinal Wholemounts and Brn3a Staining

Two weeks after IR injury, retinas were dissected from enucleated eyes and flattened. Retinas were immersed in PBS containing 30% sucrose for 24 h at 4 °C, then frozen for 15 min at −70 °C, blocked in PBS containing 1% bovine serum albumin and 0.5 % Triton X-100 and incubated with polyclonal goat anti-Brn3a antibody (1:100; Santa Cruz Biotechnology) for 72 h at 4 °C. After several washes, the retinas were incubated with the secondary antibody, Alexa Fluor-568–conjugated donkey anti-goat IgG antibody (1:250; Invitrogen, Carlsbad, CA) for 4 h, and subsequently washed with PBS. To evaluate the loss of RGCs, each retinal quadrant was divided into three zones—center, middle, and peripheral retina—corresponding to 1/6, 3/6, and 5/6 of the retinal radius. RGCs were counted in 32 distinct areas of 0.25 mm^2^ (two areas in the center, three areas in the middle and three areas at the periphery of each retinal quadrant) by two investigators in a blinded fashion, and the scores were averaged. The fluorescent images of the tissue were acquired and analyzed using confocal microscopy on a laser scanning microscope (LSM 510; Carl Zeiss Microscopy, Jena, Germany).

### 4.9. Statistical Analysis

Experiments were repeated at least three times. The data are presented as mean ± standard deviation (SD). Data were statistically analyzed using unpaired 2-tailed Student’s *t*-test or one-way ANOVA with post hoc Bonferroni’s test to compare experimental groups. Analyses were carried out on the SPSS software version 21.0 for Windows (SPSS Inc., Chicago, IL, USA). Values of *p* < 0.05 were considered statistically significant.

## 5. Conclusions

In conclusion, in the current study, we found that the neuroprotective effect of HDAC inhibition on RGCs was similar to previous findings and, notably, glial activation is regulated by HDAC2 more than HDAC1 in ischemic retinas. Our findings will help elucidate the relative contribution of HDACs to retinal glial cell activation and target the therapeutic potential of isoform-specific HDAC inhibitors for treatment of retinal neuroinflammatory disorders.

## Figures and Tables

**Table 1:** The effect of TSA on retinal ganglion cell survival in ischemic retina.

	Cell Density (cell/mm^2^ ± SD)		
	Control	Saline-Treated IR Injury Group	TSA-Treated IR Injury Group
Center	1920 ± 289.20	1576 ± 277.69 *	1779± 234.25 *†
Middle	1714 ± 259.69	1393 ± 334.10 *	1608 ± 299.62 *†
Peripheral	1723 ± 259.75	1320 ± 299.96 *	1501 ± 284.41 *†

* *p* < 0.05, comparison between control vs. saline-treated IR injury group. † *p* < 0.05, comparison between saline-treated vs. TSA-treated IR injury group. TSA = trichostatin A; IR = ischemia-reperfusion. All comparisons were performed using one-way ANOVA with post hoc Bonferroni’s test.

## Supplementary Materials

Supplementary materials can be found at https://www.mdpi.com/1422-0067/20/20/5159/s1.
