# Supplementary material for: HDAC2 Regulates Glial Cell Activation in Ischemic Mouse Retina
Source: Int J Mol Sci. 2019 Oct 17;20(20):5159. doi: 10.3390/ijms20205159 (PMC6829428; doi:10.3390/ijms20205159)
Supplement: Supplementary file 1 [file ijms-20-05159-s001.pdf]

Supplementary

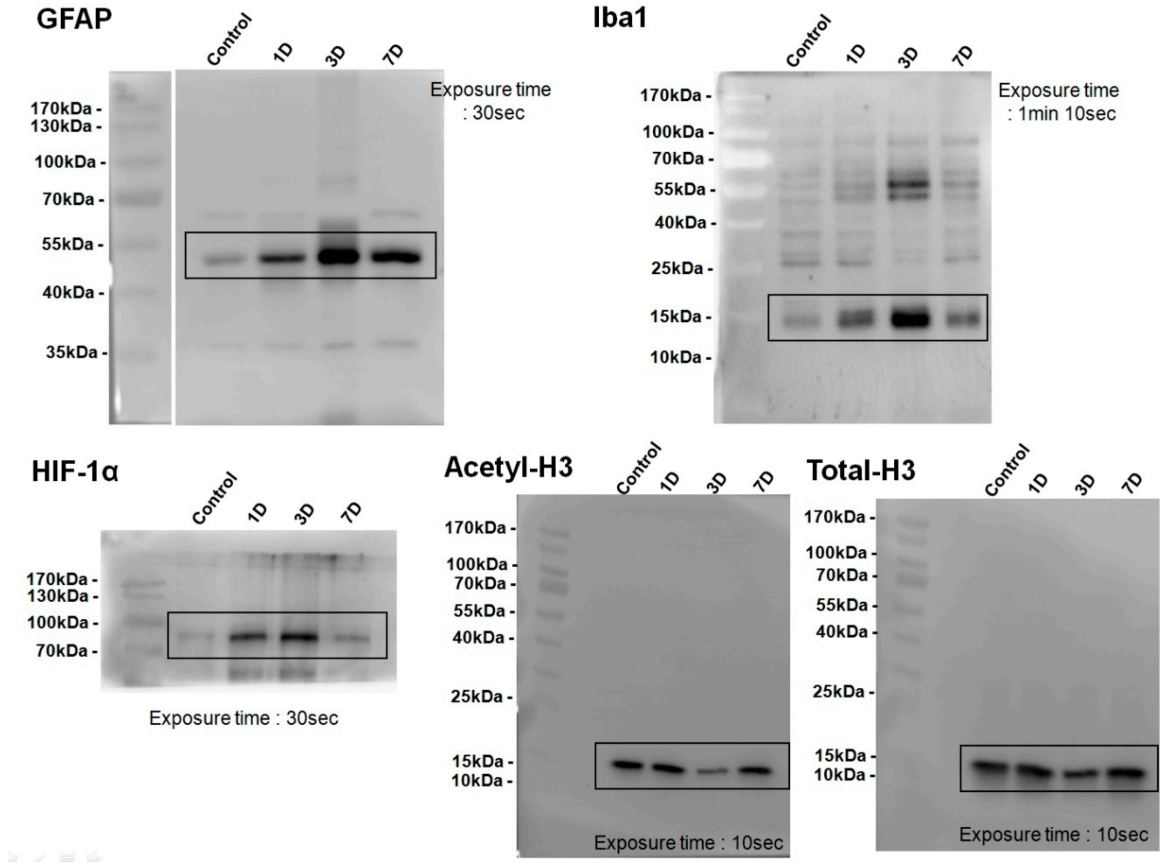

Supplementary Figure S1. Full size immunoblot image with molecular weight ladders, using anti-GFAP, anti- Iba1, anti-HIF-1α, anti-acetyl-H3, or anti-total-H3 after retinal IR injury. Cropped areas are shown in black box regions.

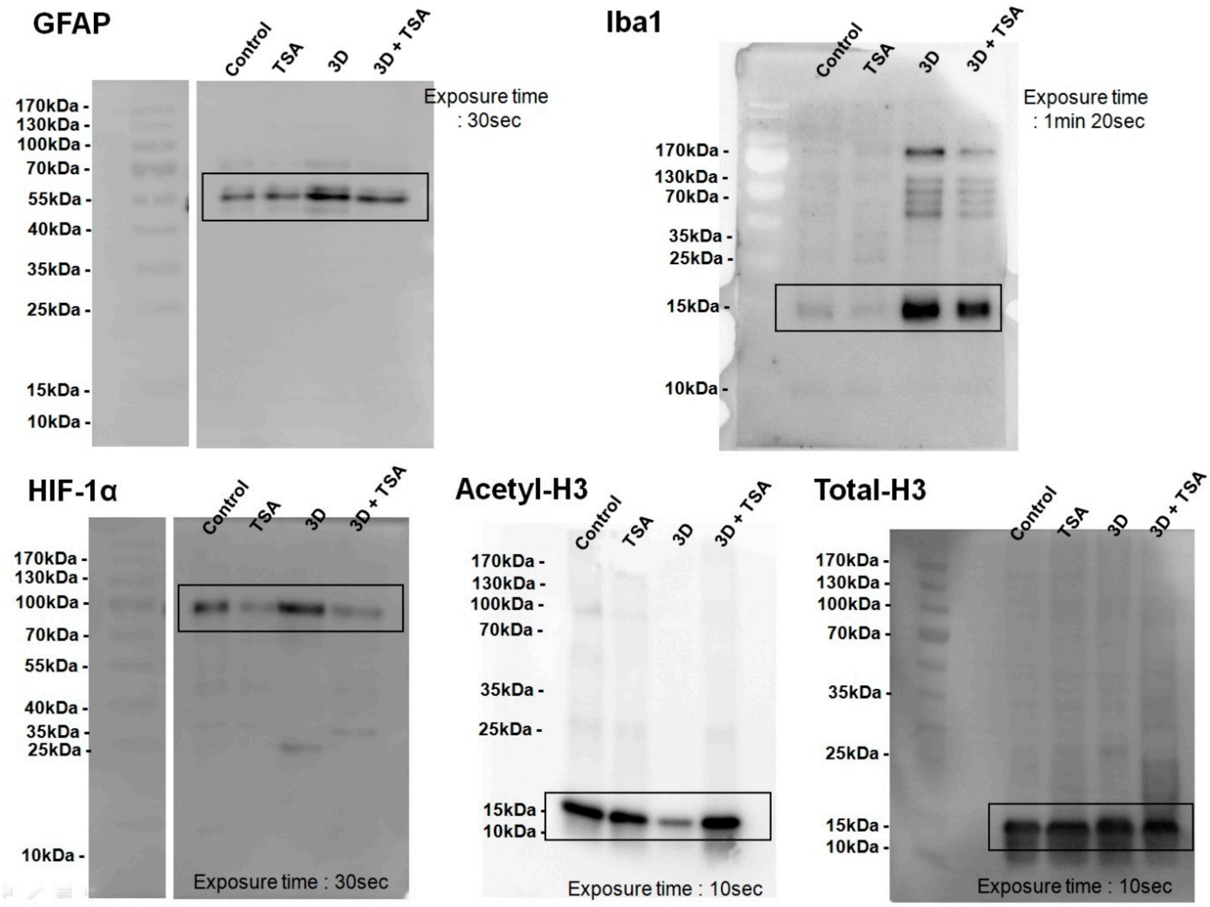

Supplementary Figure S2. Effect of TSA on expression of GFAP, Iba1, HIF-1α, and acetyl-H3 in ischemic retinas. Full size immunoblot images with molecular weight ladders are presented. Cropped areas are shown in black box regions.
